# Cytogenetic Abnormality in Exfoliated Cells of Buccal Mucosa in Head and Neck Cancer Patients in the Tunisian Population: Impact of Different Exposure Sources

**DOI:** 10.1155/2013/905252

**Published:** 2013-06-24

**Authors:** Rim Khlifi, Fatma Trabelsi-Ksibi, Amine Chakroun, Ahmed Rebai, Amel Hamza-Chaffai

**Affiliations:** ^1^Unit of Marine and Environmental Toxicology, UR 09-03, Sfax University, IPEIS, BP 1172, 3018 Sfax, Tunisia; ^2^Unit of Bioinformatics and Human Genetics, Centre of Biotechnology of Sfax, BP 1177, 3018 Sfax, Tunisia; ^3^Department of Otorhinolaryngology, Habib Bourguiba Hospital, 3000 Sfax, Tunisia

## Abstract

Chromosome/DNA instability could be one of the primary causes of malignant cell transformation. The objective of the present study was to evaluate the spontaneous genetic damages in exfoliated cells of buccal mucosa of head and neck cancer (HNC) by counting micronucleus (MN) and binucleated (BN) cells frequencies. MN and BN frequencies were significantly increased in HNC patients compared with controls (5.53 ± 3.09/1000 cells, 5.63 ± 2.99/1000 cells versus 2.36 ± 2.11/1000 cells, 3.09 ± 1.82/1000 cells, *P* < 0.001). Regarding the gender and the age, the frequencies of the MN and BN were significantly higher than those of controls (*P* < 0.01). The evaluation of the MN and BN frequencies revealed a significant increase (*P* < 0.001) in the cases in relation to the control group after controlling the risk factors (tobacco smoking and chewing and occupational exposure) of HNC. Moreover, MN and BN frequencies were significantly increased in smokers and chewers compared with nonsmokers and nonchewers among patients (*P* < 0.05). MN frequency was significantly (*P* = 0.014) different between patients occupationally exposed (6.99 ± 3.40/1000 cells) and nonexposed (4.70 ± 2.48/1000 cells) among HNC group. The logistic regression model illustrated that HNC was significantly associated with frequencies of MN (OR = 8.63, *P* < 0.0001) and BN (OR = 5.62, *P* = 0.001). Our results suggest that increased chromosome/DNA instabilities may be associated with HNC.

## 1. Introduction

Head and neck cancer (HNC) is a sentinel disease of exposure to environmental factors. The development of HNC is strongly associated with tobacco use (smoking and chewing) and alcohol consumption, as well as with the exposure to several occupational carcinogens [1–4]. Only a fraction of exposed individuals develop cancer in the head and neck region, which suggests that individual's sensitivity to mutagens is an important endogenous risk factor that significantly contributes to the development of the disease [5, 6].

HNC is a result of progressive accumulation of genetic and epigenetic alterations of HNC epithelial cells. The loss of genomic stability seems to be the main pathogenic key, appearing early in the carcinogenesis process. Genomic damage is probably the most important fundamental cause of developmental and degenerative diseases and cancer. It is also well established that genomic damage is produced by environmental exposure to genotoxins, medical procedures (e.g., radiation and chemicals), micronutrient deficiency (e.g., folate), lifestyle factors (e.g., alcohol, smoking, drugs, and stress), and genetic factors such as inherited defects in DNA metabolism and/or repair [7–12]. Hence, it is essential to perform biomonitoring with minimally invasive markers. The micronucleus (MN) trial in exfoliated cells of the buccal mucosa is a potentially excellent biomarker candidate for monitoring studies [13]. 

The micronucleus test in buccal mucosa cells is one of the less invasive methods to measure DNA damage in humans. This test was proposed in 1983 and continues to gain in popularity as a biomarker of genetic damage [14]. And its information can be used as an early warning of potential risk of developing long-term health problems [15].

The MN and BN assay with exfoliated buccal cells is a cost-effective, noninvasive method, in which the formation of anomalous cells is used as an endpoint to detect cytogenetic damage in exposed individuals. The formation of MN in dividing cells is the result of chromosome breakage due to unrepaired or misrepaired DNA lesions or chromosome malsegregation due to mitotic malfunction. These events may be induced by oxidative stress, exposure to clastogens or aneugens, genetic defects in cell cycle checkpoint and/or DNA repair genes, and deficiencies in nutrients required as cofactors in DNA metabolism and chromosome segregation machinery [16–20]. All these events can cause the formation of MN through chromosomal rearrangements, altered gene expression, or aneuploidy, effects associated with the chromosome instability phenotype often seen in cancer [17, 21].

Molecular epidemiological studies have provided evidence that individual susceptibility to cancer is mediated by genetic and environmental factors. However, the carcinogenic process is associated with increased genetic instability. To evaluate genetic instability, there are biomarkers that predict if a premalignant lesion or condition is likely to develop into an aggressive metastasizing tumor. Most cancers are monoclonal, and cytogenetic assays do provide information about the DNA damage at the level of a single but proliferating cell. The objective of the present study was to investigate the spontaneous genetic damage in exfoliated cells of the buccal mucosa of HNC patients and healthy controls by the anomalous cells (micronucleus and binucleated cells) assay with exfoliated buccal cells regarding the factors that might affect MN and BN frequencies (i.e. age, gender, smoking and alcohol drinking habits, occupational exposure, and amalgam fillings). Our study represents the first biomonitoring of these anomalous cells in HNC patients in our population.

## 2. Materials and Methods

### 2.1. Subjects Studied

The case-control study population consisted of 45 untreated cancer patients with histologically confirmed HNC and 57 cancer-free control subjects. All subjects were recruited simultaneously from residents living in the similar geographic area (Sfax). Patients were consecutively recruited between May 2012 and December 2012 at the Department of Otorhinolaryngology, Otorhinolaryngology Department, Habib Bourguiba Teaching Hospital (Sfax, Tunisia). All cases were newly diagnosed and previously untreated. Clinical characteristics including basic medical data were obtained from medical records. 

### 2.2. Questionnaire Administration

After signing the informed consents, subjects were interviewed to collect detailed information on their demographics (age and gender), alcohol drinking, tobacco smoking and chewing, amalgam fillings occupation, and occupational exposure. Lifetime consumption of tobacco and occupational exposure were also collected. The average number of cigarettes smoked per day and the total number of years of smoking were used to calculate cumulative smoking dose as “pack years” (PY = [cigarettes per day/20] × years smoked). Likewise, tobacco chewing consumption (dry snuff called *neffa)* dose was estimated as “consumption year” (CY = frequency of *neffa *consumed−kept/day × duration of year). 

### 2.3. Buccal Cells Procedure, Staining, and Scoring

The participants were asked to rinse their mouth for 1 min with 10 mL of sterilised distilled water (Braun Medical, SA) and exfoliated cells of the buccal mucosa were obtained by scraping the buccal mucosa with a wooden spatula [22, 23]. For each individual, two slides were prepared by smearing the cells immediately onto the center of clean glass slides. After applying the sample to a glass slide and drying it in the air, fixation was performed by a cold methanol-glacial acid mix (3 : 1) for 30 min. Afterwards, the glass slide was dried again, and it was then stored at room temperature until investigation of the micronuclei. Staining was carried out with 2% Giemsa solution for a period of 10 min. Afterwards, the glass slide was rinsed with aqua dest and dried in the air. The criteria of MN evaluation were those suggested by Tolbert et al. [24] and Titenko-Holland et al. [25]. 

**Figure 1:**
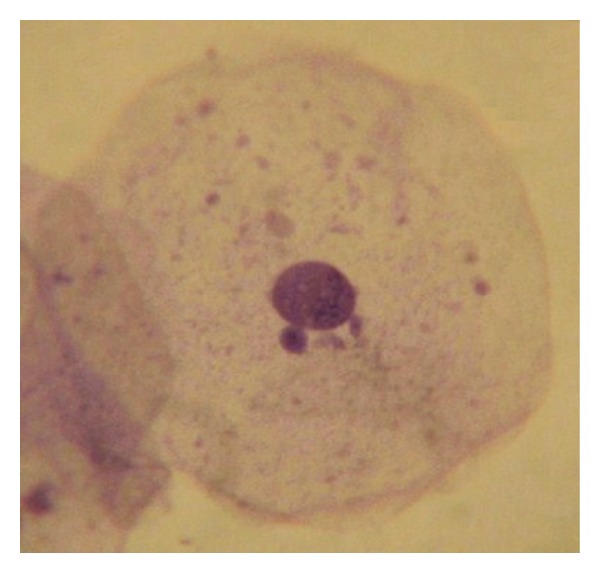
Buccal smear shows micronuclei. Pap stained. 1000X.

**Figure 2:**
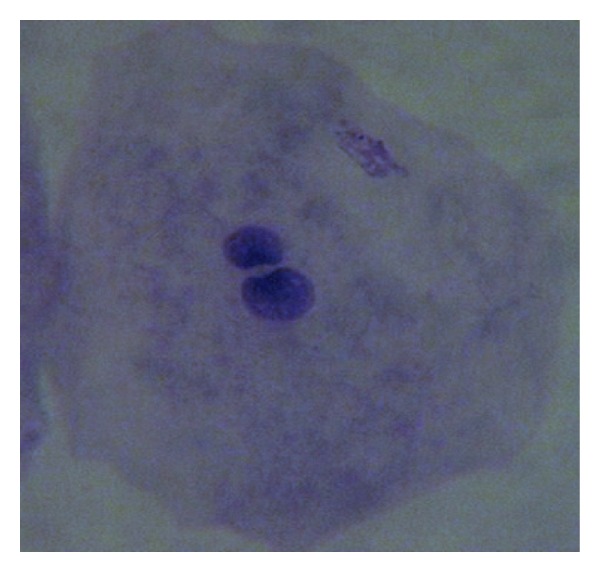
Buccal smear shows binucleated cells, Pap stained. 1000X.

**Figure 3:**
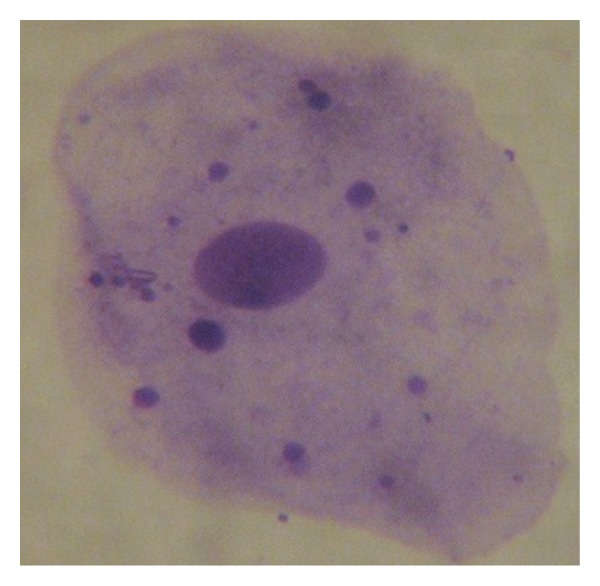
Buccal smear shows fragmented nucleus, Pap stained. 1000X.

**Figure 4:**
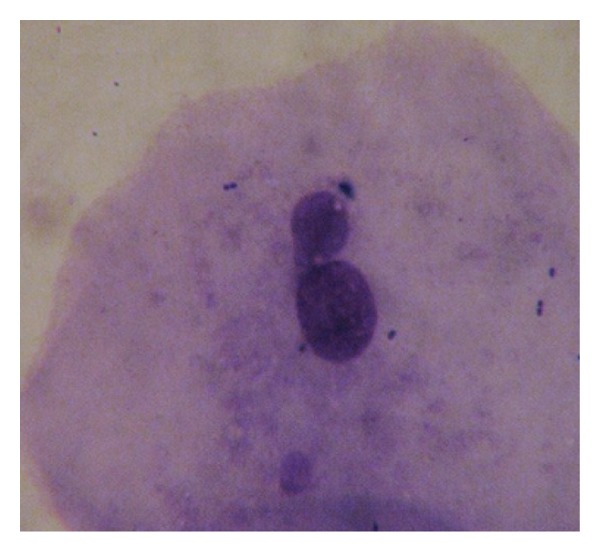
Buccal smear shows nuclei-like broken eggs, Pap stained. 1000X.

Screening for cell anomalies was performed under an oil immersion lens (2000X), followed by phase contrast microscopy for confirmation of MN according to established methods [23, 26]. At least 2000 intact epithelial cells per individual were scored to achieve the average percent of micronucleated cells. The opaque extranuclear intracytoplasmic bodies seen under oil immersion lens and phase contrast were considered micronuclei (Figure 1), whereas binucleated cells (Figure 2), fragmented nuclei (Figure 3), and nuclei-like broken eggs were not counted as MN (Figure 4).

### 2.4. Statistical Methods

The studied variable departed significantly from normality and therefore the nonparametric Mann-Whitney *U* test was applied to data. The associations between two variables were analyzed by the Spearman correlation. The chi-square test was used to compare frequencies between groups. The level of significance was taken as *P* < 0.05. To determine the effects of anomalous cell frequencies on the development of HNC, a conditional binary logistic regression model was conducted. In this model, we adjusted for age, gender, tobacco habit, occupational exposure, and alcohol drinking. In addition, the cutoffs of high-low frequency that was set at 75th percentiles of the MN and BN among the controls were used to calculate the adjusted odds ratio (OR) for HNC below and above the 75th percentiles value of MN and BN frequencies. All analyses were conducted using the Statistical Package for Social Sciences (SPSS) for Windows, version 13.0. 

## 3. Results

Descriptive characteristics of HNC cases group and the control groups are described in Table 1. Both groups were characterized for gender, age, smoking, chewing and alcohol consumption, occupation and occupational exposure, and amalgam fillings. Among patients, 75.6% were male. According to their age, patients were classified into 2 classes. 64.4% of patients aged over 55 years. The HNC cases differed significantly from the control group with respect to tobacco smoking, alcohol drinking, occupational exposure, and amalgam fillings. Thirty-one percent of HNC patients were alcohol drinkers and tobacco chewers, and sixty percent were smokers. Most of cases (64.4%) were occupationally exposed (33.3% cement workers, 20.0% farmers, and 11.1% painters). However, only 21.1% of healthy controls were occupationally exposed. A chi-square test showed that risk factors (smoking, drinking, and occupational exposure) were statistically significant between case and control groups (Table 1).

The means of MN and BN frequencies for the case and control groups are presented in Table 2, where a comparison of these values for the two cell types for all subjects is presented. The two-sample *t*-test showed that anomaly cell samples of cases were significantly higher than those of controls. The frequencies of MN and BN cells in HNC cases were higher by 2.3 and 1.8 times, respectively, than those observed in control groups. 

Table 3 shows that the frequency of the MN in exfoliated cells of buccal mucosa differs between men and women within only the control groups (*P* = 0.040). However, the BN frequency differs between the two age groups (≤55 and >55) within the cases and control groups (*P* = 0.013 and 0.012, resp.). Moreover, MN and BN frequencies of patients were mostly significantly higher than those of controls (*P* < 0.01) after controlling the gender and the age. 

The Spearman correlation coefficient matrix of selected cell anomalies number in HNC cases was also studied (Table 4). A significant positive correlation was noted between MN frequency and cases of smokers (*r* = 0.311, *P* < 0.01) and chewers (*r* = 0.227, *P* < 0.05). The correlation between chewer cases (*r* = 0.364, *P* < 0.05) and filling material amalgam (*r* = 0.430, *P* < 0.01) and BN frequency was also found. 

To evaluate possible associations between the environmental exposure and the incidence of genetic damage in buccal cells, the MN and BN data were classified according to the sampled risk factors of HNC (Table 5). The evaluation of the frequency of the MN and BN in exfoliated cells of buccal mucosa revealed a significant increase (*P* < 0.001 and <0.05, resp.) in the cases in relation to the control group after controlling these risk factors (tobacco smoking and chewing, alcohol drinking, amalgam fillings, and occupational exposure) of HNC. There was a significant difference between smokers and nonsmokers in the frequency of BN among patient and control groups (*P* = 0.046 and 0.045, resp.). Furthermore, a significant difference between smokers and nonsmokers was found in the MN frequency among HNC patients (*P* = 0.030). Among patient group, the MN frequency of participants occupationally exposed (6.99/1000 cells) was significantly higher than that of nonexposed (4.70/1000 cells) (*P* = 0.014). There was no significant difference between participants with filling material amalgam and those without filling material amalgam, in the frequency of anomalous cells in cancer patients and healthy controls. 

The 75th percentiles of the controls' MN and BN frequencies were used (as the cutoffs to assign the study subjects into either the low- or high-frequency group) in order to estimate the crude odds ratios for HNC (Table 6). High MN frequency demonstrated a strong association with HNC (OR = 8.63, *P* < 0.0001), as did high BN frequency (OR = 5.62, *P* = 0.001) (Table 6). The variables that showed significant means differences between cases and controls were included in the conditional logistic regression analysis to identify the adjusted OR and confidence interval, as seen in Table 5. After adjusting for age and gender, the results were significant for MN and BN frequencies for HNC (OR = 18.13, *P* < 0.0001 and OR = 4.91, *P* = 0.004, resp.). However, after adjusting for age, gender, tobacco habits, occupational exposure, and alcohol drinking, the results were significant only for MN frequency (OR = 69.06, *P* = 0.001) (Table 6).

## 4. Discussion

Micronuclei have been proposed as a good biomarker to assess cytogenetic damage in biomonitoring studies. Many investigations have shown an association between risk factors for squamous cell carcinomas in the head and neck and the MN rate [27–31]. In the present study, HNC patients' numbers of MN and BN cells were significantly higher than those of controls (Table 2). These observations indicate genetic damage [32], which correlates with cancer of the oral cavity [33]. Our findings were in accordance with several recent case-control studies; they revealed that spontaneous genetic damage in exfoliated buccal cell MN frequencies of patients was significantly higher than that of controls and thus genetic instability appeared to exist in exfoliated buccal cell MN frequencies of HNC patients [34–38]. Another study of HNC patients undergoing radiotherapy observed increased genomic instability in somatic cells (exfoliated buccal epithelia) in comparison to healthy control subjects [39–41].

Gender and age are considered the most important demographic variables affecting the MN index. In the present study, the MN frequency in exfoliated cells of buccal mucosa of women was significantly higher than that of men among control groups (*P* = 0.040). Furthermore, cases' cell anomaly numbers of MN and BN were mostly significantly higher than those of controls (*P* < 0.05) after controlling the gender and the age (Table 3). Our results were in accordance with Cao et al.'s [36] study that revealed a significant difference of MN frequency between oral cancer patients and controls after controlling the gender and the age. Although many studies report the age and sex of the study subjects, only a fraction of these studies were able to establish a statistically significant effect by gender [42, 43] or by age [44–46]. In another study of healthy subjects from Poland, neither age nor sex was significantly associated with MN [47]. The increase of MN with age is likely due to a combination of factors which include (a) the cumulative effect of acquired mutations in genes involved in DNA repair, chromosome segregation, and cell cycle checkpoint and (b) numerical and structural aberrations in chromosomes caused by exposure to endogenous and exogenous genotoxins [48]. The increase in MN frequency in females can be accounted for by the greater tendency of the X chromosome to be lost as an MN relative to other chromosomes and to the fact that females have two copies of the chromosome compared to only one in males [48]. 

Although mass screening to identify precancers with malignant potential for oral cancer is not feasible [12], some biomarkers are essential for this identification. Some oral oncologists report that buccal cell changes are used to categorize the molecular mechanism associated with tobacco use and oral cancer and are hence considered as good biomarkers for early detection of oral cancer [49]. This is because buccal epithelial cells are first to be exposed and interact with the xenobiotic compounds such as tobacco (nicotine), which in turn induces the frequency of micronuclei under the influence of saliva [44, 50]. Our results, showing MN and BN cell frequencies of cases of buccal mucosa which were mostly significantly higher than those of controls for tobacco smoking and chewing risk factors of HNC (Table 5). In previous studies, a significantly higher frequency of anomalous buccal cells was revealed in smokers compared to nonsmokers [47, 51, 52]. However, in the present study no significant difference between smokers and nonsmokers in the frequency of MN was observed in cancer patients and healthy controls (Table 5). Recently, Ladeira et al. [53] found that smoking habits did not influence the frequency of the biomarkers, whereas alcohol consumption only influenced the MN frequency in controls (*P* = 0.011), with drinkers showing higher mean values. Yet other publications report no difference between smokers and nonsmokers [28]. Stich and Rosin [49] concluded that neither alcohol nor smoking, alone, increased MN frequency in buccal cells, but a combination of both resulted in a significant elevation in micronucleated cells in the buccal mucosa. However, the synergism between alcohol consumption and tobacco has not been observed to act upon all biomarkers and, in several studies of lifestyle factors, it was difficult to differentiate the effect of alcohol from that of smoking [13]. 

In the last 20 years the MN assay has been applied to evaluate chromosomal damage for biological monitoring of human populations occupationally exposed to a variety of mutagenic and carcinogenic chemical or physical agents. Many studies report a statistically significant elevation of MN levels in exposed individuals compared to control groups [45, 54–56], and many other studies report changes that were not statistically significant [57, 58]. In this study, anomalous cell (MN and BN) frequencies of cases of buccal mucosa were mostly significantly higher than those of controls for participants occupationally exposed (Table 5). Our observation is in agreement with several earlier reports [53, 55, 59–65]. Long-term exposures to chemicals and exposure conditions, such as those to which some workers are subjected for occupational reasons, are suspected to be associated with genotoxic effects, which can be evaluated by analysis of biomarkers. In reviews of the literature, Bolognesi [66], Bull et al. [67], and Holland et al. [13] concluded that occupational exposure was associated with an increase in DNA damage, but a number of studies failed to detect excess cytogenetic damage compared with nonexposed populations. Although several cytogenetic biomonitoring studies on workers exposed to chemical products have been reported, there is only limited information on this topic from developing countries where chemical products have been widely used over the years. 

Regarding a connection between amalgam fillings status and anomalies rate, it became clear that particularly the amalgam fillings correlated with the MN and BN numbers (Tables 4 and 5). At the same time, an unfavourable effect of the filling material composite could be observed in comparison with amalgam which is a clinical confirmation of the *in vitro* studies of Schweikel et al. [68, 69]. Those authors found *in vitro* that extracts from five common dental composites by a majority displayed mutagenic effects (MN induction in fibroblast cell line V79), and they demanded a replacement of the mutagenic composite parts by biocompatible substances. This is of relevance as synthetic dental materials have (in)-direct contact with the oral mucosa [70] and unpolymerized monomers (e.g., 2-hydroxyethyl methacrylate, bisphenol-A-glycidyl methacrylate, and methyl methacrylate) may affect these. However, regarding amalgam fillings it is known that they form a potential permanent source of organic-bonded mercury (mainly methyl mercury) which possesses toxic characteristics and in whose formation bacteria of the oral cavity (streptococci) are involved [71].

It is not clear whether the increase of MN and BN frequencies is due to cancer development. It is possible that MN and BN frequencies before the onset of cancer were normal, but they increased after the beginning of the disease and could be a consequence of the disease status. The increased frequency of micronucleus in cancer patients may reflect an increased susceptibility of these subjects to chromosomal damage. This hypothesis was strongly supported by a case-control study nested in a large European cohort, which followed up healthy subjects examined with cytogenetic assays for cancer incidence [72]. Findings from this study showed that the higher frequency of chromosomal damage observed in subjects who develop cancer thereafter was largely due to a higher individual susceptibility rather than to the exposure to carcinogens [73]. Several factors could explain cancer predictivity of MN, for example, environmental exposure to genotoxic agents, lifestyle factors, micronutrient deficiency, and genetic factors [74].

To summarize, we believe that micronuclei assay is an effective technique adopted for rapid risk assessment of HNC. At this time it remains unclear whether elevated frequencies of MN and BN in certain tissue, such as oral epithelia, would be predictive of increased risk of future cancer, say, only for oral cavity, limited to upper digestive tract epithelia, or may be projected for various cancers in other parts of the body. Our results indicate that the increased MN and BN frequencies in exfoliated cells of the buccal mucosa of patients with HNC may reflect genomic instability or deficiency of DNA repair capacity. Thus, MN assay may be performed in exfoliated cells of the buccal mucosa as an indicator of genomic instability relevant to head and neck tumorigenesis. Further, these results suggest that increased chromosome/DNA instabilities may be associated with HNC.

## Figures and Tables

**Table 1:** Comparison of demographic and lifestyle characteristics between HNC cases and controls.

Characteristics	Controls no. (%)	Patients no. (%)	P*
Sample size	*n* = 57	*n* = 45	
Gender			0.004
Male	30 (52.6)	34 (75.6)	
Female	27 (47.4)	11 (24.4)	
Age (years)			NS
Mean ± SD	53.19 ± 15.48	59.42 ± 15.85	
≤55	25 (43.4)	16 (35.6)	
>55	32 (52.6)	29 (64.4)	
Tobacco smoking			<0.001
No	44 (77.2)	18 (40.0)	
Yes	13 (22.8)	27 (60.0)	
Mean ± SD (PY)^a^	3.64 ± 9.13	38.93 ± 35.37^c^	
Tobacco chewing			NS
No	48 (84.2)	31 (68.9)	
Yes	9 (15.8)	14 (31.1)	
Mean ± SD (CY)^b^	1.70 ± 4.57	13.91 ± 20.44^c^	
Alcohol drinking			<0.001
No	57 (100.0)	31 (68.9)	
Yes	0 (0.0)	14 (31.1)	
Occupational exposure			<0.001
No	45 (78.9)	16 (35.6)	
Yes	12 (21.1)	29 (64.4)	
Mean ± SD (year)	2.82 ± 6.81	18.87 ± 15.56^c^	
Occupation			NS
Cement worker	7 (12.3)	15 (33.3)	
Farmer	5 (8.8)	9 (20.0)	
Painter	5 (8.8)	5 (11.1)	
Amalgam fillings			0.001
No	38 (66.7)	27 (60.0)	
Yes	19 (33.3)	18 (40.0)	

*Two-sided chi-square test. ^a^PY: smoking index expressed as the number of packs of 20 tobacco cigarettes per day for 1 year.

^b^CY: consumption year = frequency of *neffa *consumed−kept/day × duration of year.

^c^ *P* < 0.001.

**Table 2:** Anomalous cell frequency in buccal mucosa exfoliated cells (/1000 cells) of HNC patients and healthy controls.

	Controls				Cases			
	Mean ± SD	Range	Median	5–95th percentiles	Mean ± SD	Range	Median	5–95th percentiles
Anomalies								
MNC	2.36 ± 2.11	0–9	1.37	0.51–7.20	5.53 ± 3.09*	2–14	4.33	2.03–11.70
BNC	3.09 ± 1.82	1–8	3.00	0.99–7.10	5.63 ± 2.99*	1–16	5.00	2.76–13.05

SD: standard deviation; MN: cells with micronuclei; BN: binucleated cells.

*Significantly different compared with the control group, *P* < 0.001.

**Table 3:** The average anomalous cells in buccal mucosa exfoliated cells in HNC patients and healthy controls, by gender and age variables.

	Anomalous cell (mean ± SD/1000 cells)			
	MN		BN	
	Controls	Cases	Controls	Cases
Gender				
Male	1.65 ± 1.33	3.43 ± 1.33**	3.22 ± 1.62	4.48 ± 1.57*
Female	2.99 ± 2.48	3.79 ± 0.75**	2.97 ± 2.00	5.62 ± 1.64*
*P* value	0.040	NS	NS	NS
Age (year)				
≤55	2.26 ± 1.97	6.45 ± 3.48**	3.76 ± 1.75	5.01 ± 1.47**
>55	2.43 ± 2.24	5.00 ± 2.69**	2.56 ± 1.72	5.95 ± 2.75**
*P* value	NS	NS	0.012	0.013

**Significantly different compared with the control group, *P* < 0.001.

*Significantly different compared with the control group, *P* < 0.01.

NS: nonsignificant.

**Table 4:** The Spearman correlation analysis of cell anomalies of HNC patients.

	Anomalies		Characteristics			
	MN	BN	Smoking	Alcohol	Chewing	Amalgam
Anomalies						
MN						
BN	0.339					
Characteristics						
Smoking	0.311**	0.227*				
Alcohol	0.149	0.097	0.440**			
Chewing	0.174	0.364*	−0.320	0.285		
Amalgam	0.212	0.430**	−0.122	−0.164	−0.062	
Occupational exposure	0.184	0.298	0.000	−0.075	−0.208	0.073

*P < 0.05.

**P < 0.01.

**Table 5:** Anomalous cell frequency observed in 1000 cells depending on HNC risk factors.

	Anomalous cell **(**mean ± SD)			
	MN		BN	
	Controls	Cases	Controls	Cases
Tobacco smoking				
No	2.46 ± 2.24	5.43 ± 2.95**	3.95 ± 1.90	5.32 ± 2.13**
Yes	1.97 ± 1.63	5.65 ± 3.24**	2.83 ± 1.73	6.09 ± 3.97**
*P* value	NS	NS	0.046	0.045
Alcohol drinking				
No	2.36 ± 2.11	5.72 ± 3.22**	3.09 ± 1.82	5.93 ± 3.35**
Yes	—	5.07 ± 2.36	—	4.95 ± 2.00
*P*-value	—	NS	—	NS
Tobacco chewing				
No	1.70 ± 1.33	4.85 ± 2.35**	2.98 ± 1.25	5.37 ± 3.27**
Yes	2.48 ± 2.22	5.76 ± 3.25**	3.10 ± 1.91	6.35 ± 1.29**
*P*-value	NS	0.030	NS	NS
Occupational exposure				
No	1.82 ± 1.56	4.70 ± 2.48**	2.99 ± 1.85	5.36 ± 3.06**
Yes	2.50 ± 2.23	6.99 ± 3.40**	3.45 ± 1.69	5.78 ± 2.24**
*P*-value	NS	0.014	NS	NS
Amalgam fillings				
No	2.12 ± 1.79	5.21 ± 2.81**	3.20 ± 1.78	5.59 ± 2.88**
Yes	2.84 ± 2.63	5.98 ± 3.31*	2.87 ± 1.78	5.69 ± 3.22*
*P*-value	NS	NS	NS	NS

**Significantly different compared with the control group, *P* < 0.001.

*Significantly different compared with the control group, *P* < 0.01.

NS: nonsignificant.

**Table 6:** Logistic regression of anomalous cell (mean ± SD/1000 cells) for HNC risk.

Anomalous cell (mean)	Controls/patients	Crude OR (95% CI)	*P*	Adjusted OR^b^ (95% CI)	*P*	Adjusted OR^c^ (95% CI)	*P*
MN^a^							
Low (≤3.00)	44/12	1.00 (reference)		1.00 (reference)		1.00 (reference)	
High (>3.00)	13/33	8.63 (3.24–22.98)	<0.0001	18.13 (5.00–65.62)	<0.0001	69.06 (5.50–185.12)	0.001
BN^a^							
Low (≤4.10)	45/17	1.00 (reference)		1.00 (reference)		1.00 (reference)	
High (>4.10)	12/28	5.62 (2.07–15.24)	0.001	4.91 (1.60–14.66)	0.004	1.87 (0.31–11.11)	NS

OR: odds ratio, CI: confidence interval.

^a^The 75th percentiles of the controls' MN and BN frequencies were used as the cutoffs to assign the study subjects to either the low-frequency group or the high-frequency group.

^b^Adjusted for age and gender.

^c^Adjusted for age, gender, tobacco (smoking and chewing), alcohol, and occupational exposure.

## Abbreviations

BN: Binucleated cells
HNC: Head and neck cancer
MN: Micronucleus.
